# Longitudinal analysis of caloric requirements in critically ill trauma patients: a retrospective cohort study

**DOI:** 10.1007/s00068-023-02429-z

**Published:** 2024-02-14

**Authors:** Christian Tibor Josef Magyar, Beat Schnüriger, Nastassja Köhn, Dominik A. Jakob, Daniel Candinas, Matthias Haenggi, Tobias Haltmeier

**Affiliations:** 1 Department of Visceral Surgery and Medicine, Inselspital, Bern University Hospital, University of Bern, Bern, Switzerland; 2 Department of Visceral Surgery, Cantonal Hospital Aarau, Aarau, Switzerland; 3 Department of Emergency Medicine, Inselspital, Bern University Hospital, University of Bern, Bern, Switzerland; 4 Department of Intensive Care Medicine, Inselspital, Bern University Hospital, University of Bern, Bern, Switzerland

**Keywords:** Multiple Trauma, Critical Illness, Nutritional Support, Calorimetry, Indirect, Treatment Outcome

## Abstract

**Purpose:**

Nutrition is of paramount importance in critically ill trauma patients. However, adequate supply is difficult to achieve, as caloric requirements are unknown. This study investigated caloric requirements over time, based on indirect calorimetry, in critically ill trauma patients.

**Methods:**

Retrospective cohort study at a tertiary trauma center including critically ill trauma patients who underwent indirect calorimetry 2012–2019. Caloric requirements were assessed as resting energy expenditure (REE) during the intensive care unit stay up to 28 days and analyzed in patient-clustered linear regression analysis.

**Results:**

A total of 129 patients were included. Median REE per day was 2376 kcal. The caloric intake did not meet REE at any time with a median daily deficit of 1167 kcal. In univariable analysis, ISS was not significantly associated with REE over time (RC 0.03, p = 0.600). Multivariable analysis revealed a significant REE increase (RC 0.62, p < 0.001) and subsequent decrease (RC – 0.03, p < 0.001) over time. Age < 65 years (RC 2.07, p = 0.018), male sex (RC 4.38, p < 0.001), and BMI ≥ 35 kg/m^2^ (RC 6.94, p < 0.001) were identified as independent predictors for higher REE over time. Severe head trauma was associated with lower REE over time (RC – 2.10, p = 0.030).

**Conclusion:**

In critically ill trauma patients, caloric requirements significantly increased and subsequently decreased over time. Younger age, male sex and higher BMI were identified as independent predictors for higher caloric requirements, whereas severe head trauma was associated with lower caloric requirements over time. These results support the use of IC and will help to adjust nutritional support in critically ill trauma patients.

**Supplementary Information:**

The online version contains supplementary material available at 10.1007/s00068-023-02429-z.

## Background

Adequate nutritional support is of paramount importance in critically ill patients, including trauma patients. Previous studies reported significant nutritional deficits in trauma patients [1, 2]. Accordingly, total body fat, glycogen, and protein have been shown to significantly decrease over time in critically ill trauma patients [3]. Infectious complications [4, 5], prolonged Intensive Care Unit (ICU) length of stay (LOS) [5], prolonged mechanical ventilation [5, 6], and higher mortality [6, 7] have been reported to be associated with underfeeding in this patient population. According to the current guidelines of the Society of Critical Care Medicine (SCCM) and American Society for Parenteral and Enteral Nutrition (ASPEN) [8], as well as the European Society for Clinical Nutrition and Metabolism (ESPEN) [9], nutritional therapy should be provided 24–48 h after the onset of critical illness and be increased to full support within 7 days.

On the other hand, overfeeding may also lead to complications such as liver dysfunction, renal functional impairment, refeeding syndrome, infectious complications, more ventilator days, longer LOS, and increased mortality [10–14]. However, the assessment of caloric requirements is difficult, as patient-, disease-, and treatment-related factors all affect requirements. These factors include age, sex, body composition and temperature, brain activity, use of sedatives, paralytic agents, and stimulants [15], as well as surgical interventions [16]. Adding to the problem, calculated caloric requirements based on predictive equations are inaccurate, as shown in previous publications [17, 18].

Indirect calorimetry (IC) is considered the current gold standard to estimated caloric requirements [19–21]. Multiple factors, including patient characteristics, disease-related stress, sepsis, the amount of nutrition, and medical treatments affect energy expenditure in critically ill patients [19, 22, 23]. Previous studies have reported and discussed energy expenditure as measured per IC in critically ill patients [14, 23–26]. However, in these studies, energy expenditure was measured for only 14 days [14, 24, 25]. Furthermore, patients with varying medical and surgical conditions were included, not taking into account the different patient characteristics [27] and metabolic response of trauma and non-trauma patients [28–30].

Considering the importance of adequate nutritional support and scarcity of specific data on energy expenditure in critically ill trauma patients, further investigation into this important topic is needed. Therefore, the current study aimed to (1) investigate the caloric requirements over time in critically ill trauma patients using IC and (2) assess the impact of clinical factors on the caloric requirements over time.

## Methods

### Patient selection and data collection

This is a retrospective cohort study including critically ill trauma patients who underwent IC during their ICU stay at a tertiary care university hospital with a yearly admission of approximately 600 severely injured trauma patients. The study was performed in accordance with the ethical standards as laid down in the 1964 Declaration of Helsinki and was approved by the Ethics Committee of the Swiss Canton of Bern (ID 2018-01744). Data were reported according to the STrengthening the Reporting of OBservational studies in Epidemiology (STROBE) guidelines, which are supported by the Enhancing the QUAlity and Transparency Of health Research (EQUATOR) network.

Adult trauma patients (age ≥ 16 years) admitted to the ICU between January 1, 2012, and December 31, 2019, with one or more IC performed during the ICU stay, were included in the study.

Patients and injury characteristics, treatment modalities, caloric requirements based on IC, caloric intake, and clinical outcome variables were extracted from electronic medical records. Injury severity was assessed using the Abbreviated Injury Scale (AIS) and Injury Severity Score (ISS). Both, ISS and AIS were extracted from the Swiss Trauma Registry and electronic medical records. Severe injury was defined as AIS scores ≥ 3.

### Caloric requirements by indirect calorimetry

Caloric requirements were assessed over time, i.e., up to four weeks after ICU admission. Resting energy expenditure (REE), as a measure for caloric requirements, was measured by IC. In IC, REE is calculated from the measured oxygen consumption (VO_2_) and carbon dioxide production (VCO_2_), using Weir’s equation [REE (kcal/d) = 1.44 (3.9*VO_2_ + 1.1*VCO_2_)]. The GE Carescape B850 Monitor System E-COVX Module (GE HealthCare, Chicago, IL, USA) was used to measure REE. The decision to measure the REE was made selectively by the attending physician once patients had reached a clinically stable phase, provided that their oxygen level on the ventilator was below 70%. Typically, measurements were conducted during night shifts (11 pm–7 am) and lasted for at least 2 h. During the measurement, ventilator settings remained unchanged. The moving average of the REE signal was recorded in a patient data management system (Clinisoft, GE, Anandic Medical Systems AG, Switzerland) and verified for accuracy through visual inspection. The median REE value was subsequently calculated for each measurement period.

### Caloric intake and deficit

The caloric intake, including total parenteral nutrition (TPN) and enteral nutrition (EN), was recorded during 24 h and documented in electronic patient records. In every patient, the caloric deficit was calculated by subtracting the overall median intake from the overall median REE. To adjust for the different ICU LOS of the patients included, the median caloric deficit was dived by the number of ICU days, providing the daily median caloric deficit.

### Clinical outcomes

The following clinical outcome variables were recorded: Infectious complications, including urinary tract infection, catheter related blood stream infection, surgical site infection (superficial, deep, organ space), ventilator-associated pneumonia, and sepsis (according to clinical diagnosis in patient charts), in-hospital and 30-day mortality, ICU LOS, total hospital LOS, and ventilator days. Infectious complications were defined according to the Centers for Disease Control and Prevention (CDC) National Healthcare Safety Network (NHSN) guidelines. The Acute Physiology and Chronic Health Evaluation (APACHE) II scoring system was used to assess the severity of organ dysfunction.

### Statistical analysis

Normality of distribution was assessed using histograms, skewness, and the Shapiro–Wilk test. Categorical variables were reported as numbers and percentages, continuous variables as medians and interquartile ranges (IQR). Changes of REE over time and the effect of clinical factors on REE over time were assessed in a univariable and multivariable, population-based, patient-clustered linear regression analysis. Regression models with different fitted time variables modelling were evaluated. Based on the lowest *p*-value and optimal likelihood ratio, a curvilinear regression was chosen for the final model. Different values for age and BMI were assessed. In the final analysis, cutoff values with the highest coefficient were used. Effect sizes were reported as regression coefficients (RC) with 95% confidence intervals (95% CI). Multivariate analysis was performed including all variables with *p* value < 0.10 in univariable analysis. The effect of the median daily caloric deficit on clinical outcomes was assessed in univariable logistic regression analysis. For visualization, changes of REE over time were plotted using LOESS curve fitting. The median caloric deficit the day prior to the performance of IC vs. the day after was compared using Wilcoxon signed-rank test. *P*-values of 0.05 or less were considered statistically significant. Statistical assistance was provided by the Clinical Trials Unit of the University of Bern. Analysis was performed using SPSS Statistics (IBM Corporation, Armonk, NY, USA) and R Studio software (R Studio, Inc, Boston, MA, USA).

## Results

### Patient population and treatment characteristics

A total of 129 critically ill trauma patients with at least one indirect calorimetry performed during the ICU stay were included in the study. Patient, injury, and treatment characteristics are outlined in Table 1. Patients were predominantly male (79.8%) with a median age of 56 years (IQR 38–67). Most frequent comorbidities were heart failure (27.9%), diabetes mellitus (14.0%), pulmonary disease (12.4%), chronic kidney disease (8.5%), and chronic liver disease (6.2%).

**Table 1:** Patient, injury, and treatment characteristics

	Number (%) or median (IQR)	
	n = 129	
Patient characteristics		
Age [years]^b^	56 (38–67)	
Male sex^a^	103 (79.8)	
BMI [kg/m^2^]^b^	26.0 (23.9–29.2)	
Heart failure^a^	36 (27.9)	
Diabetes mellitus^a^	18 (14.0)	
Pulmonary disease^a^	16 (12.4)	
Chronic kidney disease^a^	11 (8.5)	
Chronic liver disease^a^	8 (6.2)	
APACHE at admission^b^	23 (19–27)	
Injury characteristics		
Blunt trauma^a^	123 (95.3)	
Injury Severity Score (ISS)^b^	36 (29–45)	
Injury Severity Score > 15	128 (99.2)	
Severe head trauma (AIS ≥ 3)^a^	77 (59.7)	
Severe face trauma (AIS ≥ 3)^a^	12 (9.3)	
Severe chest trauma (AIS ≥ 3)^a^	99 (76.7)	
Severe abdominal trauma (AIS ≥ 3)^a^	35 (27.1)	
Sever pelvic or extremity trauma (AIS ≥ 3)^a^	60 (46.5)	
Severe external trauma (AIS ≥ 3)^a^	2 (1.6)	
Vital signs and laboratory values on ICU admission		
Min. SBP [mmHg]^b^	63 (58–74)	
Max. heart rate [bpm]^b^	93 (82–107)	
Max. temperature [°C]^b^	37.3 (36.5–37.8)	
Max. lactate [mmol/L]^b^	2.8 (1.6–4.4)	
Max. white blood count [G/L]^b^	10.1 (7.3–15)	
Min. hemoglobin [g/L]^b^	98 (84–113)	
Surgical interventions		
Orthopedic^a^	100 (77.5)	
Neurosurgical^a^	49 (38.0)	
Laparotomy^a^	17 (13.2)	
Thoracotomy^a^	16 (12.4)	
Blood product transfusion		
PRBC transfusion [250 mL units]	96 (74.4)^a^	4 (2–8)^b^
Plasma transfusion [280 mL units]	30 (23.3)^a^	2 (2–5)^b^
Platelets transfusion [250 mL units]	32 (24.8)^a^	2 (1–5)^b^

*BMI* Body Mass Index, *APACHE* Acute Physiology and Chronic Health Evaluation score, *AIS* Abbreviated Injury Scale, *SBP* systolic blood pressure, *PRBC* packed red blood cells

^a^Number (%)

^b^Median (interquartile range)

Most patients suffered from blunt trauma (95.3%). The median ISS was 36 (IQR 29–45) with severe head, face, chest, abdominal, pelvic or extremity, and external trauma in 59.7%, 9.3%, 76.7%, 27.1%, 46.5%, and 1.6%, respectively.

Orthopedic and neurosurgical procedures were performed in 77.5% and 38.0%, respectively. Laparotomies were conducted in 13.2% and thoracotomies in 12.4%. A total of 97 (75.2%) patients received blood product transfusions. Median units of packed red blood cells (PRBC), plasma, and platelets transfused were 4 (IQR 2–8), 2 (IQR 2–5), and 2 (IQR 1–5), respectively.

### Resting energy expenditure

**Fig. 1:**
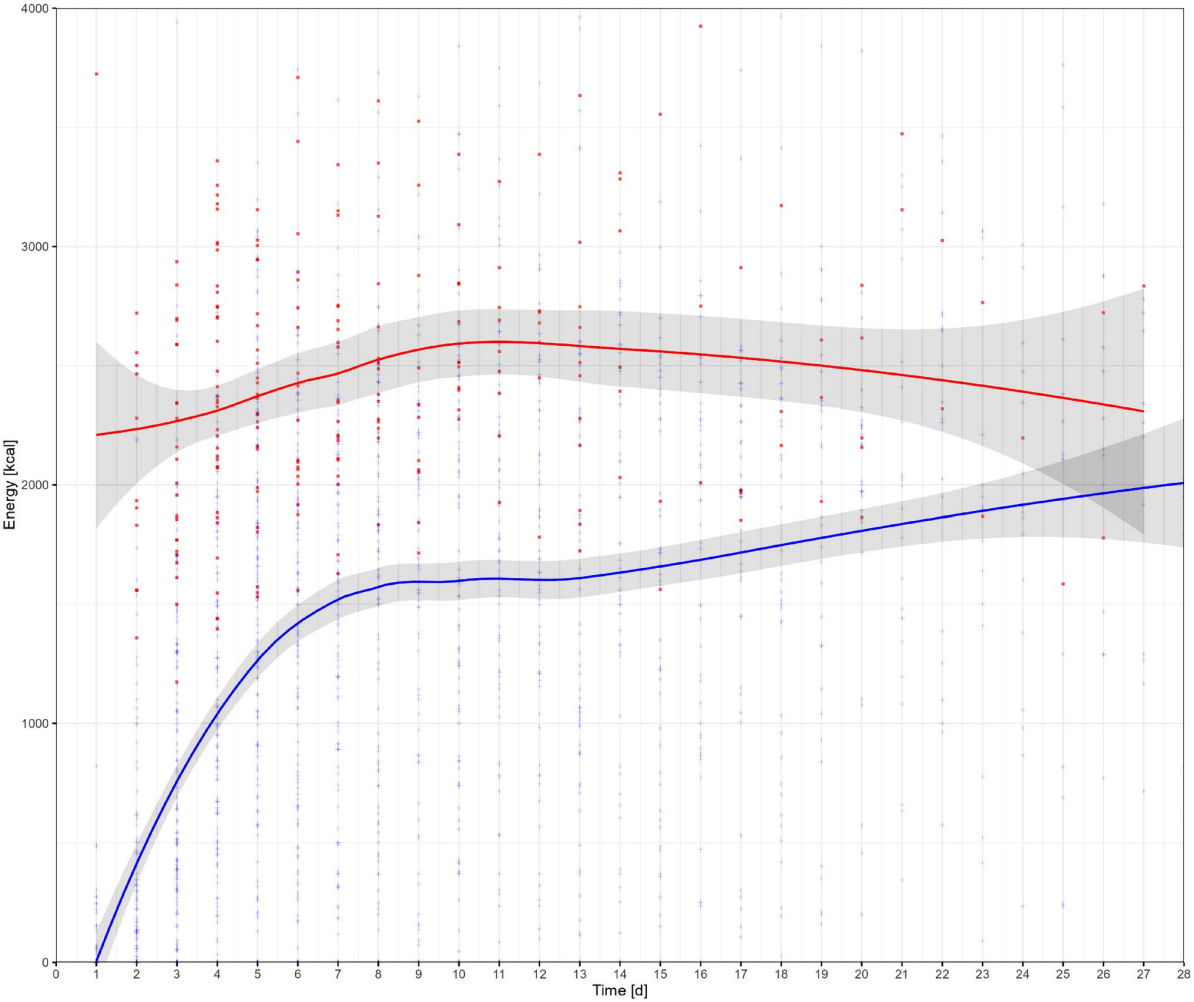
Resting energy expenditure [red] as measured per indirect calorimetry and caloric intake [blue] in critically ill trauma patients within 28 days from ICU admission. LOESS curve fitting with 95% confidence intervals. *d* days, *kcal* kilocalories

The median REE per day was 2376 kcal (IQR 2036–2748) (Table 2). REE significantly increased and then decreased over the ICU stay, which is reflected by the RC of 0.62 for study days and -0.03 for squared study days in multivariable regression analysis (Table 4). The transition from REE increase to decrease was observed on day 11 of the ICU stay (Fig. 1).

**Table 2:** Resting energy expenditure, caloric intake, and caloric deficit per day

	Number of patients (%)	Median kcal per day (IQR)
Resting Energy Expenditure	129 (100)	2376 (2036–2748)
Caloric intake		
Total parenteral nutrition	16 (12)	1312 (794–1727)
Enteral nutrition	129 (100)	1105 (230–1971)
Total nutritional intake^a^	129 (100)	1275 (322–2032)
Caloric deficit	129 (100)	-1167 (-1732 to -631)

^a^Enteral and parenteral nutrition

**Fig. 2:**
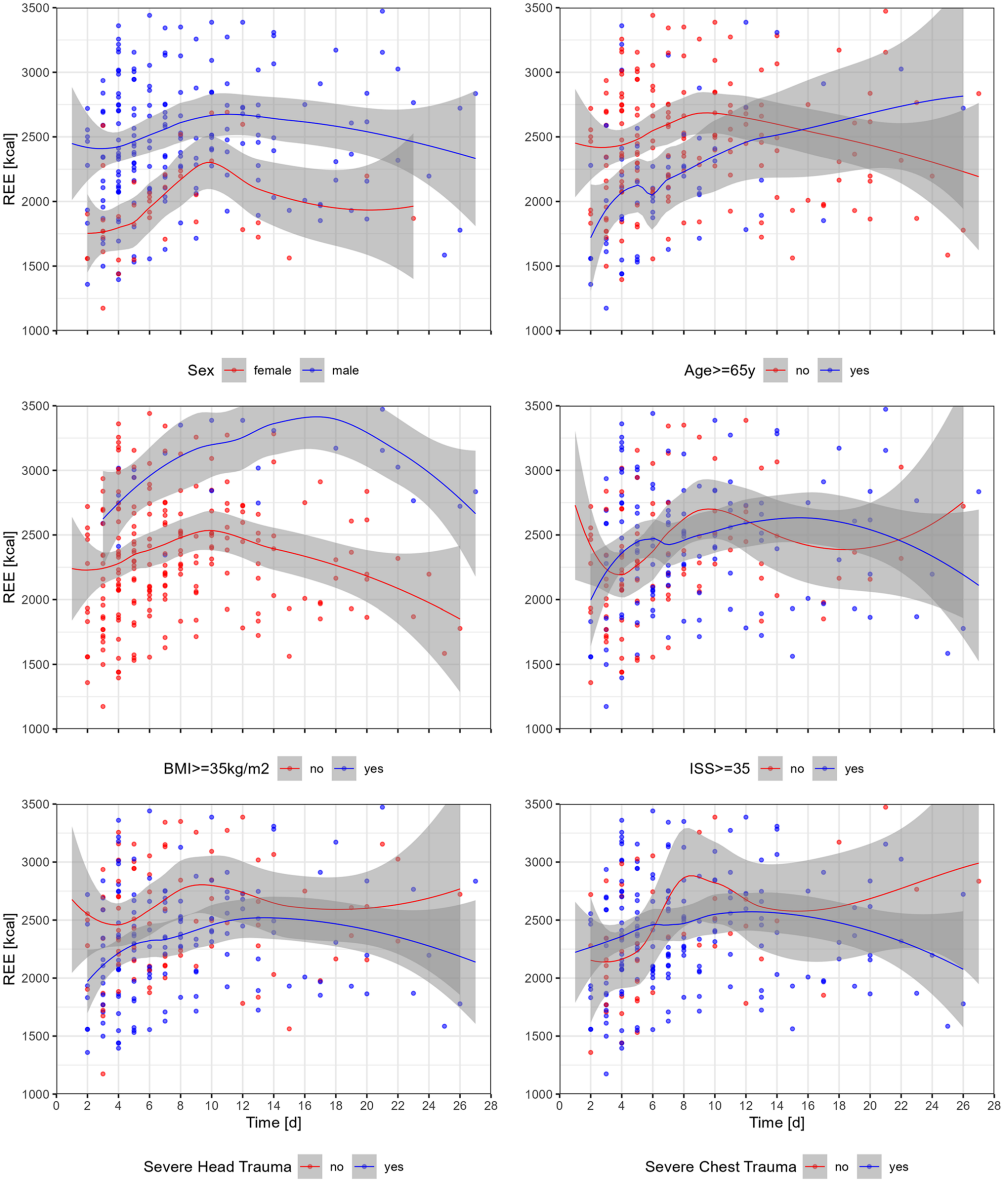
Resting energy expenditure (REE) as measured per indirect calorimetry in critically ill trauma patients within 28 days from ICU admission, stratified by patient and injury characteristics (A-F). LOESS curve fitting with 95% confidence intervals. *Age and BMI* cutoff values with highest coefficient in regression analysis. Severe head trauma and chest trauma defined as Abbreviate Injury Score ≥ 3 in respective body regions. ISS cutoff chosen based on median. *d* days, *kcal* kilocalories, *BMI* body mass index, *ISS* Injury Severity Score

In univariable regression analysis, male sex, age < 65 years, BMI ≥ 35 kg/m^2^, PRBC transfusion, and laparoscopy were associated with significantly higher REE over time, whereas severe head trauma and severe external trauma were related to significantly lower REE over time. ISS and co-morbidities were not significantly associated REE over time. (Table 3, Fig. 2) Due to the low occurrence of 1.6%, severe external trauma was not entered in further analysis.

**Table 3:** Univariable analysis of effect of patient, injury, and treatment characteristics on resting energy expenditure (in steps of + 100 kcal)

	Coefficient	95% CI	p value
Patient characteristics			
Age	-0.08	-0.15 to -0.01	0.035
Age < 65 years	6.33	3.70 to 8.97	0.006
Male sex	5.92	3.46 to 8.39	< 0.001
BMI [kg/m^2^]	0.18	-0.01 to 0.37	0.057
BMI ≥ 35 kg/m^2^	3.68	0.91 to 6.45	0.009
Heart failure	-2.77	-5.90 to 0.37	0.084
Diabetes mellitus	-1.86	-5.58 to 1.86	0.300
Pulmonary disease	0.64	-3.10 to 4.38	0.700
Chronic kidney disease	-2.06	-6.66 to 2.54	0.400
Chronic liver disease	0.88	-2.47 to 4.24	0.606
APACHE at admission	-0.10	-0.30 to 0.10	0.339
Injury characteristics			
Injury severity score	0.03	-0.08 to 0.14	0.600
Severe head trauma (AIS ≥ 3)	-3.50	-6.65 to -0.42	0.027
Severe face trauma (AIS ≥ 3)	-1.50	-4.71 to 1.72	0.400
Severe chest trauma (AIS ≥ 3)	2.80	-0.03 to 5.67	0.054
Severe abdominal trauma (AIS ≥ 3)	1.40	-1.54 to 4.27	0.400
Severe pelvic and extremity trauma (AIS ≥ 3)	0.19	-2.91 to 3.30	> 0.900
Severe external trauma (AIS ≥ 3)	-21.00	-22.77 to -19.15	< 0.001
Treatment characteristics			
PRBC transfusion [250 mL units]	0.21	0.01 to 0.41	0.039
Plasma transfusion [280 mL units]	-0.06	-0.19 to 0.06	0.300
Platelet transfusion [250 mL units]	-0.20	-0.49 to 0.10	0.200
Laparoscopy	7.20	1.66 to 12.79	0.012
Laparotomy	2.70	-1.22 to 6.66	0.200
Thoracotomy	0.10	-3.31 to 3.52	> 0.900
Neurosurgery	0.47	-2.74 to 3.67	0.800
Orthopedic surgery	-0.12	-2.77 to 2.53	> 0.900

Univariable population based (patient clustered) linear regression analysis

*CI* confidence interval, *BMI* Body Mass Index, *APACHE* Acute Physiology and Chronic Health Evaluation score, *AIS* Abbreviated Injury Scale, *PRBC* packed red blood cells

Multivariable regression analysis revealed age ≤ 65 years (RC 2.07, 95% CI 0.35–3.79, p = 0.018), male sex (RC 4.38, 95% CI 2.32–6.44, p < 0.001), and BMI ≥ 35 kg/m^2^ (RC 6.94, 95% CI 5.16–8.73, p < 0.001) as independent predictors for higher REE over time. Severe head trauma (RC -2.10, 95% CI -4.00 to -0.20, p = 0.030) was independently associated with lower REE over time (Table 4).

**Table 4:** Multivariable analysis of effect of patient- and treatment characteristics on resting energy expenditure (in steps of + 100 kcal)

	Coefficient	95% CI (lower–upper)	p value
Age < 65 years	2.07	0.35 to 3.79	0.018
Male sex	4.38	2.32 to 6.44	< 0.001
BMI ≥ 35 kg/m^2^	6.94	5.16 to 8.73	< 0.001
Heart failure	-1.58	-3.24 to 0.08	0.061
Severe head trauma (AIS ≥ 3)	-2.10	-4.00 to -2.00	0.030
Severe chest trauma (AIS ≥ 3)	0.26	1.00 to 1.94	0.763
PRBC transfusion [250 mL units]	-0.03	-0.14 to 0.08	0.592
Surgery, laparoscopy	0.77	1.00 to 3.86	0.626
Day	0.62	0.27 to 0.97	0.001
Day^2^	-0.03	-0.04 to -0.01	< 0.001

Multivariable population based (patient clustered) linear regression analysis

*CI* confidence interval, *BMI* Body Mass Index, *AIS* Abbreviated Injury Scale, *PRBC* packed red blood cells

### Caloric intake and deficit

EN was administered in all patients included with a median daily intake of 1105 kcal (IQR 230–1971). TPN was given to 16 patients (12.0%) with a median daily intake of 1312 kcal (IQR 794–1727). The median total caloric intake per day was 1275 kcal (IQR 322–2032). The median caloric deficit per day was -1167 kcal (IQR -1732 to -631) (Table 2). The median caloric deficit was significantly higher the day prior to the performance of IC vs. the day after (-1118 kcal [IQR -1841 to -419] vs. -777 kcal [IQR  -1494 to -133], p < 0.001). As shown in Fig. 1, the caloric intake increased over time, but never met the calculated requirements.

### Clinical outcomes

Median ICU and total hospital LOS were 12 days (IQR 7–18) and 15 days (IQR 8–24), respectively. The majority of patients (82.9%) were transferred to another hospital or rehabilitation. In-hospital and 30-day mortality was 8.6% and 10.9%, respectively. Detailed clinical outcomes including infectious complications are outlined in Supplemental Table 1. No significant effect of the median caloric deficit per day on clinical outcomes was found in univariable regression analysis (Supplemental Table 2).

## Discussion

The aim of this retrospective cohort study was to investigate caloric requirements over time, as measured by indirect calorimetry, and the effect of clinical factors on requirements, in critically ill trauma patients.

Caloric requirements significantly increased and then decreased over time. Younger age, male sex, and higher BMI were identified as independent predictors for higher caloric requirements over time, whereas severe head trauma was independently associated with lower requirements over time. Injury severity was not significantly associated with caloric requirements over time.

The findings of this study support use of IC in critically ill trauma patients. As caloric requirements change over time, the caloric intake must be adjusted, especially in patients with a longer ICU stay. Furthermore, the knowledge of the clinical factors associated with caloric requirements identified in the current study will help to guide nutrition in critically ill trauma patients.

The impact of the use of IC in the current study is reflected by the lower caloric deficit the day after IC compared to the day before. The use of IC in critically ill patients is recommended by the current guideline of the SCCM and ASPEN [8], as well as the ESPEN [9]. A meta-analysis published in 2021 reported that IC-guided energy delivery significantly reduces short-term mortality [31]. However, the quality of the studies included in this meta-analysis was moderate and IC-guided nutrition was not associated with significantly better outcomes in the individual randomized controlled trials [24, 25, 32–35]. Thus, further investigation regarding the potential benefit of IC on clinical outcomes is warranted.

Earlier work has investigated caloric requirements over time based on IC in critically ill patients [24, 25, 35–37]. However, the proportion of trauma patients included in these studies was low, ranging from 9 to 21% [24, 35, 36]. Another study investigating energy expenditure based on IC over time included patients with TBI only [38]. In contrast, the current study investigated critically ill trauma patients exclusively. This is of importance, as characteristics of critically ill trauma patients differ substantially from critically ill non-trauma patients. It has been show that trauma patients are younger, more frequently male, require more often mechanical ventilation, and have lower APACHE scores compared to non-trauma patients [27], all of which may affect nutritional requirements.

No evidence for a caloric ebb phase, as described in 1942 by Cuthbertson et al. [39], was found in the current study. This finding are in line with newer concepts of energy production from endogenous sources and anabolic resistance early in the clinical course [20, 40]. It is thought that the endogenous energy production during the acute disease phase approximates energy demands and cannot be suppressed by exogenous nutrition. Feeding during the early phase of acute illness may therefore result in a relative overfeeding [41]. In this context, randomized trials have shown that higher nutritional support did not benefit critically ill patients and was associated with higher morbidity [42, 43]. Currently, a gradual increase in caloric intake during acute phase is recommended by the SCCM-ASPEN and ESPEN guidelines [8, 9].

In the current study, the caloric intake gradually increased over time, but never met the requirements. This corresponds with the results of a previous prospective study investigating caloric requirements over time in trauma patients. Of note, in this previous study, caloric requirements were calculated based on predictive equations, but not IC [2]. The prevalence of malnutrition in critically ill adult patients has been reported as 38–78% [44]. Multiple causes for insufficient nutrition in critically ill patients have been described, including delayed initiation and slow advancement of nutrition, under-prescription, incomplete delivery, and frequent interruption of nutrition. Interruptions may be caused by diagnostic tests, surgical procedures, gastrointestinal intolerance, feeding tube problems, and routine nursing procedures [45]. In one prospective study, only 26% of causes for interrupted enteral nutrition were found to be avoidable [46].

Previous studies have reported worse outcomes in critically ill patients with an energy deficit [36, 47]. In the current study, no significant association between the caloric deficit and clinical outcomes was found (Supplemental Table 2). The non-significant association of the caloric deficit and clinical outcomes in the current study may be explained by the smaller and decreasing caloric deficit over time (Fig. 1). Furthermore, this study included trauma patients only, whereas the other studies investigated critically ill patients with different medical and surgical conditions. This is of significance, as trauma patients are not expected to be in a malnourished state on admission and may therefore better tolerate an initial energy deficit.

Interestingly, injury severity was not significantly associated with caloric requirements over time in the current study. Two older studies reported similar findings, i.e., no correlation of the ISS and energy expenditure [48] and metabolic abnormalities [49]. In another older analysis, the ISS correlated with higher energy expenditure. However, only 15 trauma patients were included in this study [50]. Thus, considering the results of the current study and previous work, the ISS seems not to affect caloric requirements.

The strengths of this study are the use of IC as a standard of care and assessment of caloric requirements and intake over the ICU stay, up to four weeks. Apart from the usual restrictions of a retrospective study, this study has some inherent limitations. First, IC was performed at the treating intensivists discretion, which may have led to a selection bias. Second, additional small caloric intakes such as propofol or glucose infusions were not recorded. Third, while the results of this study may be generalizable to a larger trauma population, the small number of patients with severe external injuries, including burns, prohibits an extrapolation for this specific group of trauma patients. This is of importance, as burn injuries are known to be associated with increased caloric requirements [51]. Fourth, the number of patients included in the current study was relatively small, which is reflected by the wide confidence intervals in multivariable analysis. Fifth, as REE was measured in mechanically ventilated patients, the results cannot be directly extrapolated to non-ventilated patients. Nevertheless, the predictors for caloric requirements identified in this study are likely to be valid for non-ventilated patients, too.

In critically ill trauma patients, caloric requirements significantly increased and subsequently decreased over time. The caloric intake gradually increased over time, but never met the calculated requirements. Younger age, male sex and higher BMI were identified as independent predictors for higher caloric requirements, whereas severe head trauma was associated with lower caloric requirements over time. In contrast, the injury severity was not significantly associated with caloric requirements over time. These results support the use of IC and will help to adjust nutritional support in critically ill trauma patients.

### Supplementary Information

Below is the link to the electronic supplementary material.

Supplementary file1 (DOCX 17 KB)
