# Supplementary material for: Longitudinal analysis of caloric requirements in critically ill trauma patients: a retrospective cohort study
Source: Eur J Trauma Emerg Surg. 2024 Feb 14;50(3):913–23. doi: 10.1007/s00068-023-02429-z (PMC11249493; doi:10.1007/s00068-023-02429-z)
Supplement: Supplementary file 1 — Supplementary file1 (DOCX 17 KB) [file 68_2023_2429_MOESM1_ESM.docx]

| **Supplemental Table 1** Clinical outcomes | |
| --- | --- |
|  | Number (%) or median (IQR) |
|  | n=129 |
| Urinary tract infection^*^ | 4 (3.1) |
| Catheter related bloodstream infection^*^ | 10 (7.8) |
| Surgical site infection overall^*^ | 13 (10.1) |
| SSI superficial^*^ | 3 (2.3) |
| SSI deep^*^ | 10 (7.8) |
| SSI organ-space^*^ | 2 (1.6) |
| Wound dehiscence^*^ | 2 (1.6) |
| Ventilator associated pneumonia^*^ | 28 (21.7) |
| Sepsis^*^ | 10 (7.8) |
| ICU length of stay [days] ^†^ | 12 (7-18) |
| Hospital length of stay [days] ^†^ | 15 (8-24) |
| In-hospital mortality^*^ | 11 (8.6) |
| 30-day mortality^*^ | 14 (10.9) |
| ^*^Number (%), †median (interquartile range).  ICU: intensive care unit; SSI: surgical site infections. | |

| **Supplemental Table 2** Univariable analysis of effect of daily caloric deficit on clinical outcomes | | |
| --- | --- | --- |
|  | Odds ratio (95% CI) | p-value |
| Urinary tract infection | 0.95 (0.79 – 1.15) | 0.603 |
| Catheter related bloodstream infection | 0.99 (0.90 – 1.09) | 0.992 |
| Surgical site infection overall | 0.97 (0.88 – 1.07) | 0.559 |
| SSI superficial | 0.86 (0.69 – 1.09) | 0.207 |
| SSI deep | 1.01 (0.90 – 1.12) | 0.890 |
| SSI organ-space | 0.55 (0.24 – 1.25) | 0.154 |
| Wound dehiscence | 1.04 (0.85 – 1.27) | 0.719 |
| Ventilator associated pneumonia | 0.97 (0.90 – 1.03) | 0.279 |
| Sepsis | 1.01 (0.92 – 1.11) | 0.847 |
| In-hospital mortality | 1.00 (0.92 – 1.09) | 0.999 |
| 30-day mortality | 1.00 (0.93 – 1.08) | 0.987 |
| One-year mortality | 0.99 (0.93 – 1.06) | 0.820 |
| Univariable logistic regression analysis. The daily caloric deficit was entered in steps of 100 kcal.  CI: confidence interval; SSI: surgical site infection. | | |
